# Biosynthesis and assembly of hydrogenase [NiFe]-cofactor: recent advances and perspectives

**DOI:** 10.1093/mtomcs/mfaf015

**Published:** 2025-05-24

**Authors:** R Gary Sawers, Maximilian Hardelt, Alexander Haase, Dorothea Lubek

**Affiliations:** Institute for Microbiology, Martin Luther University Halle-Wittenberg, Halle (Saale), Germany; Institute for Microbiology, Martin Luther University Halle-Wittenberg, Halle (Saale), Germany; Institute for Microbiology, Martin Luther University Halle-Wittenberg, Halle (Saale), Germany; Institute for Microbiology, Martin Luther University Halle-Wittenberg, Halle (Saale), Germany

**Keywords:** carbamoyl phosphate, carbon monoxide, cyanide, hydrogenase, hydrogenase, iron, nickel

## Abstract

The large subunit of all [NiFe]-hydrogenases in bacteria and archaea has a heterobimetallic NiFe(CN)_2_CO cofactor coordinated by four cysteine residues. The iron ion has two cyanides and a carbon monoxide as diatomic ligands. Six ancillary Hyp (ABCDEF) proteins are necessary for anaerobic synthesis of this cofactor, while under oxic conditions at least one further protein, HypX, is required for CO synthesis. The Fe(CN)_2_CO moiety of the cofactor is synthesized on a separate HypCD scaffold complex. Nickel is inserted into the apo-large subunit only after Fe(CN)_2_CO has been introduced. Recent biochemical and structural studies have significantly advanced our understanding of cofactor biosynthesis for these important metalloenzymes. Despite these gains in mechanistic insight, many questions still remain, the most pressing of which is the origin of the CO ligand in anaerobic microorganisms. This minireview provides an overview of the current status of this research field and highlights recent advances and unresolved issues.

## Graphical Abstract

**Graphical Abstract:**
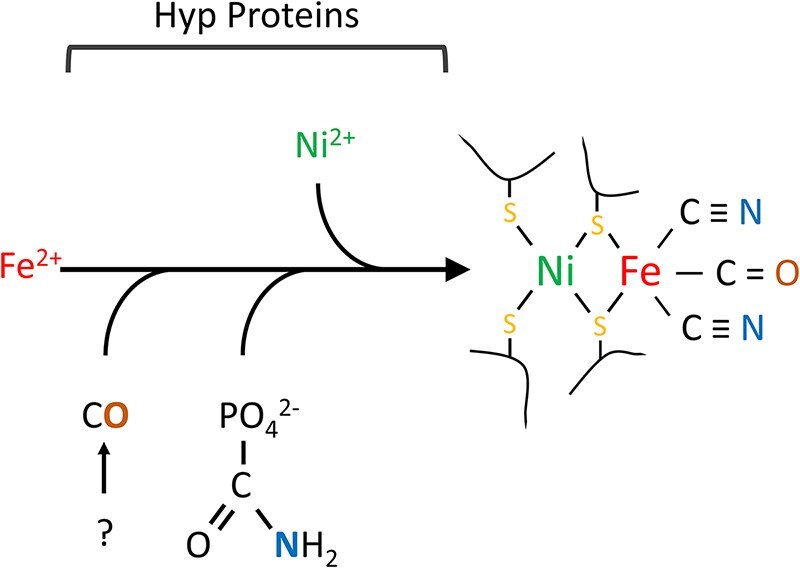
Anaerobic biosynthesis of the NiFe(CN)_2_CO-cofactor requires six Hyp proteins. The metals are delivered by nickel and iron transport systems to the Hyp machinery. The metabolic origin of the two cyanide ligands to the Fe ion is carbamoyl phosphate. The source of the CO ligand is unresolved (?), although studies on aerobic cofactor biosynthesis suggest it might originate from C1 metabolism.

## Introduction

[NiFe]-hydrogenases are found in numerous bacterial and archaeal species [1, 2]. While these enzymes, when isolated, are capable of reversible conversion of dihydrogen (H_2_) into protons and electrons, in most microorganisms, they exhibit catalytic bias, often functioning in H_2_ oxidation to provide reducing power for anaerobic respiration or pyridine nucleotide reduction [1, 3, 4]. Of course, there are important exceptions exemplified by the H_2_-evolving formate hydrogenlyase complex, which is found in both bacteria and archaea [5–7], allowing the release of excess reducing equivalents accumulated during fermentative processes in the form of the membrane-permeable gaseous product, H_2_.

**Figure 1.**
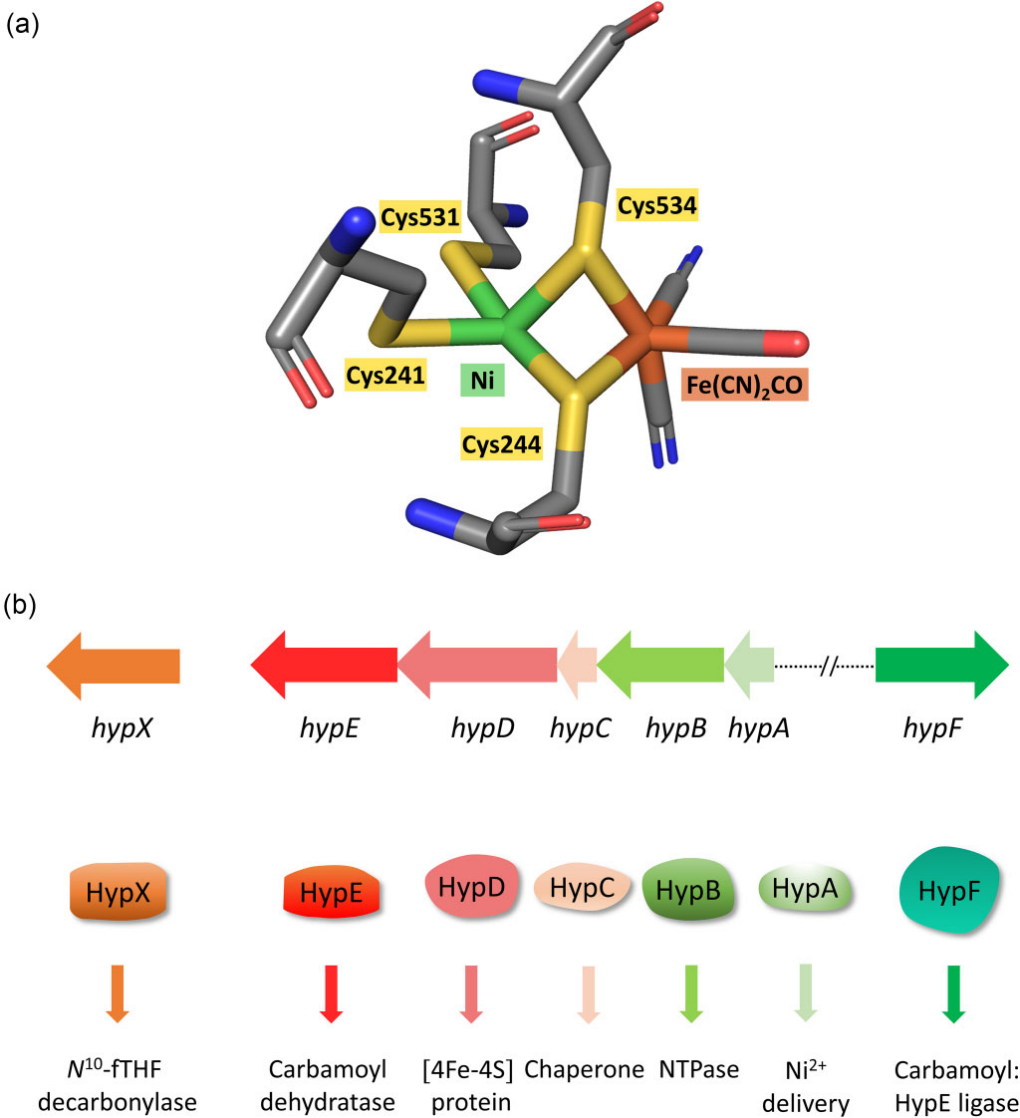
Overview of the structure and biosynthesis of the [NiFe]-cofactor. (a) Coordination of the NiFe(CN)_2_CO cofactor in the large subunit (HycE) of *Escherichia coli* hydrogenase 3. The cysteine residues that coordinate the cofactor are indicated and this structural representation was guided by PDB accession 7Z0S [7]. (b) Overview of the *hyp* genes and the function of their encoded products in [NiFe]-cofactor biosynthesis. The biochemical functions of the respective gene products are indicated. Note that the organization of the *hypA-F* genes is representative for *E. coli* and the *hypX* gene is found in *Cupriavidus necator*.

Catalytic activity of [NiFe]-hydrogenases is conferred by a NiFe(CN)_2_CO cofactor in the active site of the large subunit of these enzymes [4]. The cofactor in all [NiFe]-hydrogenases analysed to date has a nearly identical structure (Ni:Fe:CN:CO ratio is 1:1:2:1) and coordination environment within the active site (Fig. 1a). It is ligated by four conserved cysteine residues, occasionally with a selenocysteine substituting for one of the cysteine residues, which coordinate the nickel ion; two of these cysteine residues also form bridging ligands to the iron ion [4, 8]. The iron ion of the bimetallic cofactor carries one carbonyl and two cyanide ligands and these function to tune the redox state of the iron ion and it has been hypothsized that this allows the nickel to bind and heterolytically cleave H_2_ [4, 9]. This buried and shielded active site provides an environment for efficient activation of H_2_ by the cofactor. Consequently, specific pathways are required within the large subunit to allow access of the gaseous substrate/product to or from the cofactor, for proton transfer, and for electron transfer to the chain of iron–sulfur clusters found in the small subunit [10]. Considerable advances have been made in these areas of hydrogenase research in recent years and these are summarized in excellent reviews [4, 11] and so these facets of [NiFe]-hydrogenases will not be covered further here.

Rather, the focus of this minireview will be on recent advances that have been made in the study of the biosynthesis of the [NiFe]-cofactor and its subsequent insertion into the apo-active site of the catalytic subunit. The two advances made in the mid-to-late 1990s that proved to be instrumental in significantly advancing the field of [NiFe]-cofactor research were the elucidation of the first crystal structure of a [NiFe]-hydrogenase [8] and the identification by infrared (IR) spectroscopy of the carbonyl and cyanide ligands associated with the cofactor [9]. The findings of these studies immediately suggested the likely functions of the proteins encoded by a set of genes, termed *hyp* for ‘hydrogenase pleiotropy’ (Fig. 1b), which are frequently found in the neighbourhood of hydrogenase structural genes, and which, upon mutation, result in loss of all, or nearly all, hydrogenase function in the microorganism [12–17]. Consequent upon the discovery of the diatomic ligands attached to the Fe ion, several research groups using different model bacteria made rapid progress in assigning functions to the Hyp proteins required for cofactor synthesis and hydrogenase maturation [13–17]. This progress mainly resulted from their own accumulated findings over many years in which they generated and analysed mutants that had been either specifically selected, screened for, or constructed by in-frame gene deletion, along with the detailed phenotypic characterization of the mutants [reviewed in (3, 18–20)]. The key findings of those studies identified a series of steps involved in the maturation process and these will be briefly recapitulated, together with a description of more recent findings, which also highlight several still open questions in this important field of metalloprotein biochemistry.

## Step 1: Fe(CN)_2_CO synthesis

### Carbamoyl phosphate, carbamoyl: HypE ligase, and carbamoyl dehydratase

It was the hydrogenase-negative phenotype of a *pyrA* mutant of *Salmonella typhimurium* LT2 (now *S. enterica* Typhimurium) isolated by the group of Erica Barrett in the early 1980s [21] that inspired the idea to determine whether carbamoyl phosphate (CP) might be the source of the diatomic ligands of the [NiFe]-cofactor [22]; it was later realized that *pyrA* is *carB*, encoding the β-subunit of CP synthetase [23]. CP’s involvement in cofactor biosynthesis was clearly demonstrated in studies done by the Böck group [24, 25], with the unexpected outcome that CP was the metabolic source of only the two cyanide ligands, but not that of the carbonyl ligand [26–28]. As will be discussed below, the metabolic origin of the CO ligand during anaerobic hydrogenase maturation is still unresolved.

Analysis of the primary sequence of HypF (carbamoyl: HypE ligase) identified the presence of a *C*-terminal *O*-carbamoyl transferase domain, as well as a putative acyl-transferase domain [29]. Subsequent characterization of purified HypF identified CP and adenosine triphosphate (ATP) as substrates [24]. The specific interaction between HypF and HypE led to the proposal and subsequent demonstration that HypF indeed has a carbamoyl transferase activity. The HypF enzyme has been proposed to generate initially carbamoyl-AMP as activated intermediate, which concomitantly stabilizes carbamate, and then uses this as substrate for the *S*-carbamoylation of the *C*-terminal cysteine residue of HypE, generating a thiocarboxamide modification [25, 30]. HypE (carbamoyl dehydratase) then dehydrates the thiocarboxamide in an ATP-dependent reaction that generates a thiocyanate moiety attached to the cysteine. The cyanyl group is then ready for reductive transfer to the iron ion that is proposed to be coordinated by a HypC-HypD scaffold complex [30].

### The HypCD scaffold complex synthesizes Fe(CN)_2_CO

**Figure 2.**
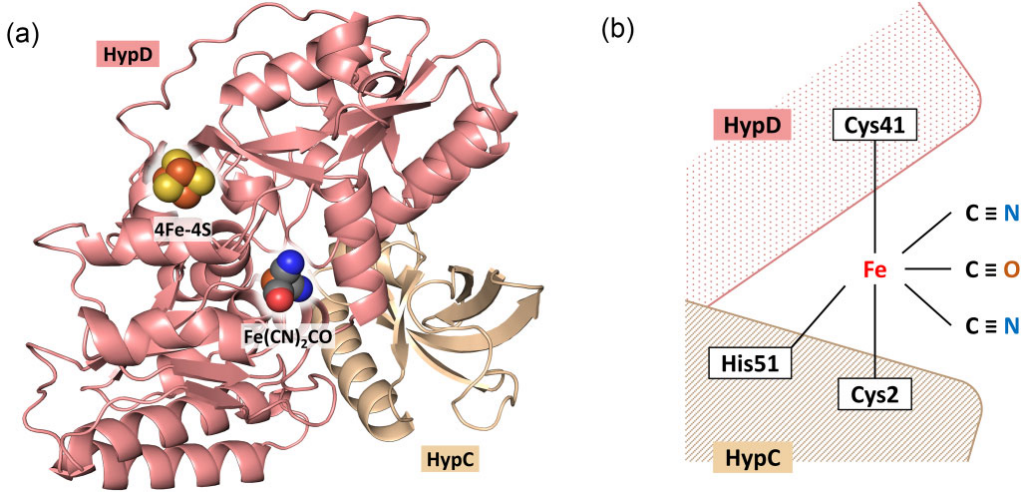
Structural prediction of the holo-HypCD complex of *E. coli*. (a) AlphaFold3 simulation of the *E. coli* HypCD complex. The ipTM and pTM scores of the prediction are 0.78 and 0.81, respectively. AlphaFill was used to add the [4Fe–4S]-cluster. The Fe(CN)_2_CO group was added manually. (b) Schematic representation of the proposed coordination of the Fe(CN)_2_CO moiety by the *E. coli* HypCD complex.

The first indication that the iron component of the cofactor might be synthesized on a separate scaffold, and not directly in the active site of the hydrogenase large subunit, was suggested when the apo-large subunit of the *E. coli* hydrogenase 3 was unexpectedly isolated in association with the small maturation protein, HypC [31]. On the basis of that finding, HypC was suggested to have a ‘chaperone-like’ function, delivering a component of the cofactor after its synthesis. Subsequent analyses identified that HypC also interacts with the [4Fe–4S] cluster-containing protein, HypD, forming a scaffold complex [32]. This central protein-protein interaction, occurring during [NiFe]-cofactor biogenesis, was shown to be commonly found during hydrogenase maturation in other microorganisms [33, 34] and ultimately led to the clear demonstration for hydrogenase maturation in *C. necator* (formerly *Ralstonia eutropha*) [35] and *E. coli* [36] that the Fe(CN)_2_CO moiety of the cofactor is assembled on this HypCD scaffold complex (Fig. 2).

While the core of the scaffold complex is HypD bound to HypC, or its homologues [29, 37], early studies showed that the carbamoyl dehydratase, HypE, only transiently interacts with the complex, delivering the cyanide ligands [30, 38]. On the other hand, the carbamoyl-HypE ligase, HypF, fails to form a stable complex *in vitro* with the HypCDE ternary complex, but it does form a stable complex with HypE when this protein is not associated with HypCD [34, 39]. However, structural analyses of initially individual Hyp proteins isolated from bacteria [39, 40], as well as from the archaeon *Thermococcus kodakarensis* [41, 42], and subsequently of HypCDE and HypEF complexes [43, 44] provided the sea change in our general understanding of the maturation process, especially on the roles of the individual Hyp proteins in cofactor biosynthesis. In particular, the structure of the HypCD complex shed light on how the Fe(CN)_2_CO moiety might be coordinated by the essential *N*-terminal cysteine residue (C2) on HypC and the highly conserved C41 residue of HypD [43] (Fig. 2b). Moreover, the presence of HypE in this complex provided first insights into how the modified *C*-terminal cysteine residue might deliver the cyanide ligands [43]. The structures of HypF and of the HypEF complex made it clear how a ‘tunnel’ within HypF shields the labile carbamate group released during CP hydrolysis until it forms a carbamoyl-adenylate, which is then delivered to the *C*-terminal thiol group on HypE [20].

### Central role of [4Fe–4S]-containing HypD in directing ligand synthesis

When HypD was initially isolated, it was immediately obvious from both its brown colour and UV-visible spectrum that it was an iron-sulfur protein [38]. As the only redox-active protein known to be involved in the synthesis of the Fe(CN)_2_CO moiety, HypD was where assembly of the group was proposed to occur [35, 36, 38, 43]. The absolutely conserved C41 (*E. coli* numbering) residue was proposed to be the site where the Fe ion initially binds, also coordinated by the *N*-terminal C2 of HypC and possibly by a conserved histidine residue (H51) on HypC (Fig. 2b). Amino acid-exchange mutagenesis of these residues revealed loss of hydrogenase enzyme activity and cofactor content, clearly supporting a role for each of these residues in the coordination of the iron group on the HypCD complex of *E. coli* [45–47]. It is currently unclear what the redox state of the iron is prior to coordination by the HypCD complex, but initially it is likely to be Fe^2+^. Whether a fifth ligand to iron is the side chain of a further stabilizing amino acid residue in the HypCD complex [see 35], or whether the site is free to coordinate CN^−^ or CO, is unresolved, but see the arguments raised in [18].

A recent study using native mass spectrometric analysis of HypCD complexes isolated anaerobically from different *E. coli* mutants has identified a modification proposed to represent the complex carrying the Fe(CN)_2_CO moiety [48]. Analysis of HypCD complexes isolated from *iscU* or *iscS* mutants, genes which encode the scaffold proteins of the ISC iron–sulfur cluster biogenesis machinery, has shown that the specific modification is absent [49]. These data suggest that the Fe ion for hydrogenase maturation on HypD may be acquired directly or indirectly from the ISC scaffold complex [50].

HypD has a number of other conserved cysteine residues that serve important functions in the protein [46]. These include the four cysteine residues that coordinate the [4Fe–4S] cluster, and which have been shown to be essential for both maturation function, manifestation of hydrogenase enzyme activity, as well as for stability of HypD [46]. Together, these findings clearly indicate that the redox activity of HypD is important in the maturation process. Two further conserved cysteine residues, C69 and C72 in *E. coli* numbering, form a thiol-disulfide exchange motif that is located between the [4Fe–4S] cluster (*E*^o^’ = −262 mV) [42, 51] and C41 and, based on data of *in vitro* studies, these have been suggested to undergo successive and reversible electron and proton transfer reactions. This led to the proposal that they may be involved in the reductive transfer of the cyanide groups from HypE to the Fe ion bound between HypD-C41 and HypC-C2 [20, 51]. Notably, however, electron transfer from the [4Fe–4S] cluster on HypD to the C69–C72 disulfide motif has not yet been directly demonstrated [38, 51]. Moreover, the physiological electron donor to the [4Fe–4S] cluster in HypD is still unresolved.

While HypC and HypD form a 1:1 complex [48], HypE and HypF form an α_2_β_2_ heterocomplex [44]. Structural analyses have demonstrated that two HypCD heterodimers bind to a HypE homodimer [20]. As the order of addition of the CN^−^ and CO ligands is also unresolved, it has been initially proposed that two successive cycles of reductive transfer of the cyanide group from the *C*-terminal cysteine of HypE occurs driven by a thiol-disulfide oxidation involving the C69/C72 residues [20]. The reduction of the disulfide bond to regenerate the thiols of C69/C72 would require electrons presumably derived from the [4Fe–4S] cluster, as well as protons; however, the origin of, and the delivery route taken by, these protons are both currently unknown.

It is also conceivable that the [4Fe–4S] cluster and the C69/C72 pair in HypD could be required for CO synthesis. Of course, this will depend very much on what the metabolic origin of the carbonyl group might be; however, if it were assumed to be CO_2_, then its direct dehydration to CO could potentially be achieved with protons and electrons delivered from the C69/C72 pair, but the driving force of −530 mV necessary to achieve this reduction [26, 52] would require an involvement of ATP hydrolysis. The discovery that the HypCD complex indeed has a weak ATPase activity could support a possible role for the activity in generation of the CO ligand [52, 53]. The HypCD complex lacks a conventional ATP-binding site, but HypD does have a Rossmann fold, which might suggest its involvement in ATP hydrolysis [46, 52]. Moreover, amino acid-exchange variants of HypD that impair this conserved motif have been analysed and these exchanges lead to an impaired ATPase activity [54]. An ATPase activity has also recently been identified to be associated with the HypCD complex isolated from *R. eutropha* (renamed *C. necator*) [53]. However, the authors suggest a plausible alternative role for ATP binding, which might be required for the transfer of the Fe(CN)_2_CO group into the apo-large subunit [53].

## Step 2: Transfer and insertion of the Fe(CN)_2_CO moiety into the apo-large subunit

As already noted, several early studies demonstrated a tight interaction between HypC proteins and the apo-large subunit of hydrogenases [31, 34, 45]. Neither HypD nor HypE was involved in these interactions and the intensity of the interaction between HypC and apo-HycE, the large subunit of hydrogenase 3, became stronger when the subsequent nickel insertion step was blocked. In contrast, the interaction could be diminished by preventing CP synthesis, in which case the HypC–HypD interaction was intensified [32]. This result also clearly established that the Fe(CN)_2_CO moiety was introduced into the active site, and HypC had to be released from its complex with the apo-large subunit prior to the addition of nickel (see also below). These proposals were further strengthened by analysis of HycE maturation in an *E. coli* mutant deficient in nickel transport [55].

The suggestion that HypC proteins shuttle between HypD and the apo-large subunit of hydrogenase gained yet further credence by using native mass spectrometry to study the transfer reaction [48]. In this case, a homologue of HypC found in *E. coli*, called HybG, and which is specifically required for maturation of H_2_-oxidizing hydrogenase 2 [37], was shown to interact exclusively with the apo-form of the hydrogenase 2 large subunit, apo-HybC, and not with the mature form of the subunit. Moreover, titration experiments revealed that HybG had a higher affinity for apo-HybC *in vitro* than for HypD, but retained its ability to enter into a 1:1 complex with either protein [48]. While that study did not examine transfer of the Fe(CN)_2_CO moiety, the differential affinity for HypD or pre-HybC that was noted has implications for the finding alluded to above, whereby prevention of CP synthesis intensified the HypC–HypD interaction, despite apo-HycE being available for interaction [32]. The implication here is that there is a further level of control *in vivo*, possibly signified by the presence of Fe lacking a full complement of diatomic ligands in the HypCD complex, which determines HypC’s (or HybG’s) interaction with its cognate apo-large subunit, and which is not recapitulated in *in vitro* experiments. Importantly, however, the findings show that HypC, or its paralogues, only interact with either HypD or their cognate apo-large subunit and do not appear to form a HypC–HypD-apo-large subunit ternary complex [45, 48, 49].

C2 on HypC is essential for interaction of the protein with apo-HycE [45]. Interestingly, one of the four cysteine residues (C241) that ultimately coordinate the [NiFe]-cofactor in HycE (Fig. 1a) is essential for the interaction of the protein with HypC, implying that this cysteine residue interacts directly or indirectly with C2 of HypC. Most likely both of these residues are required for the insertion of the Fe(CN)_2_CO moiety in a thiol-transfer reaction [18]. However, C241 is not initially ligated with the Fe group, which probably occurs at the neighbouring C244 residue [45]. Once HypC has been displaced, then C241 can coordinate the iron group, while nickel subsequently binds to C531, but only after HypC has been displaced from the complex [45]; presumably, this is aided by interaction with the HypAB complex (see below), which delivers the nickel ion. Finally, a conformational change induced by a proteolytic cleavage at the *C*-terminus closes and seals the active site [18, 20].

The question remains how HypD is displaced from the HypCD complex to allow the transfer of the Fe(CN)_2_CO moiety onto the *N*-terminally located thiol motif [18] of the apo-large subunit. HypC is clearly instrumental in this process. A recent study by the Lenz group has used a variety of approaches, including IR spectroscopy combined with structural predictions to extend the findings of a study showing Fe(CN)_2_CO group transfer to the apo-hydrogenase large subunit [56], and they provide evidence that the transfer of the Fe(CN)_2_CO moiety from HypCD to the apo-large subunit is ATP-dependent [53]. Surprisingly, however, ATP hydrolysis does not appear to be required, as the non-hydrolysable ATP analogue *β, γ-*methyleneadenosine 5′-triphosphate was also capable of facilitating group-transfer. The authors propose that ATP-binding by HypCD causes a conformational displacement of its *C*-terminally located domain to release the Fe(CN)_2_CO group from its coordination with HypD and this allows transfer of the moiety to the apo-large subunit. This data does not resolve the issue of how the transfer of the Fe group to the thiols of the apo-large subunit is achieved biochemically, but, as the authors point out, subsequent hydrolysis of the nucleotide might induce transfer of the group, priming HypC for a further maturation cycle [53].

## Step 3: Fidelity of nickel insertion

One of the first mutants to be isolated with a defect in total hydrogenase activity could be phenotypically complemented by adding high, sub-toxic, concentrations of NiCl_2_ (low mM) to the growth medium [57–59]. The mutant proved to have a mutation in the *hypB* gene, which encodes the nickel-binding metallochaperone and GTPase, HypB [60]. HypB is functional in cooperation with its ‘partner’ metallochaperone, HypA, and HypA paralogues. Like paralogues of HypC, those of HypA are occasionally found in bacteria that synthesize more than one hydrogenase and these proteins confer hydrogenase-specificity during the maturation process [18, 19]. Clearly, these data imply that HypA interacts directly with, and delivers nickel to, the hydrogenase large subunit already containing the Fe(CN)_2_CO moiety. This also explains why only *hypB* mutants and not mutants with mutations in *hypA* were identified in the original screen for ‘hydrogenase-negative’ mutants [58, 59]; e.g. *E. coli* has a paralogue of HypA encoded by *hybF* [18].

Details of the identification and characterization of HypA and HypB proteins from several microorganisms [61, 62] have been documented in excellent reviews [18, 19] and these features of the proteins will not be reiterated here. However, several more recent biochemical and structural studies conducted over the last few years have delivered some important new insights and mechanistic details in the areas of nickel delivery and insertion.

The first step that helps ensure only nickel and not, e.g. zinc is inserted into the active site of the hydrogenase large subunit is the transport of nickel, and this is typically induced when the bacteria synthesize their hydrogenases, and presumably helps direct nickel specifically to the HypAB complex [55, 63]. The second key step ensuring specificity for nickel is the protease that acts in the final proteolytic step, which does not catalyse peptide cleavage if another divalent cation in inadvertently inserted into the large subunit [55, 64]. Structural analyses performed by the Miki group (summarized in [20]) of the HypAB [65] and the HypA-apo- large subunit [66] complexes have been especially illuminating in elucidating these nickel-insertion steps. HypB-type GTPases are essential for nickel insertion not only into hydrogenases, but also into ureases [67]. Interestingly, in some species HypB has an additional histidine-rich nickel storage domain [68], and while most HypB proteins hydrolyze guanosine triphosphate (GTP), some archaeal HypB homologues specifically hydrolyze ATP [69]. Nevertheless, the mechanistic basis and function of these proteins remain the same, regardless of which nucleoside triphosphate (NTP) they hydrolyze [20].

Generally, HypB proteins have a GTP-binding domain and a nickel-binding region; there are, however, exceptions [20]. HypA, on the other hand, has a *N*-terminal nickel-binding domain, as well as a zinc-binding domain, the latter of which has a zinc-finger motif that coordinates a Zn^2+^ ion. This domain also exhibits conformational flexibility and can sense both nickel loading and changes in pH [70]. Based on the structural studies, a general model for nickel insertion can be formulated, whereby the ATP- or GTP-bound form of HypB binds HypA, forming an α_2_β_2_ heterotetrameric complex. Binding by NTP-bound HypB induces major conformational changes in HypA, which result in formation of a binding site with an extremely high (nM) affinity for nickel [65, 71]. Binding of nickel induces nucleotide hydrolysis by HypB, releasing nickel-bound HypA from the complex and allowing it to bind and deliver the nickel ion to the open hydrogenase active site. Despite HypA proteins from different sources being less well conserved and showing greater variability in structure compared with some other Hyp proteins, presumably because greater flexibility is required to facilitate interaction with different hydrogenase large subunits [20], the overall mechanism comprising a nickel-binding HypAB pair that undergoes nucleotide hydrolysis after cation-binding has occurred, appears to have been conserved throughout evolution. The variable level of nickel in different habitats [72] requires a system with a very high affinity and specificity for nickel, while at the same time explaining why growth in the presence of high nickel concentrations in the laboratory can bypass the need for a HypAB system [58, 59].

## Step 4: *C*-terminal cleavage and conformational closure of the active site

Proteolytic cleavage of the *C*-terminal peptide of the large subunit of [NiFe]-hydrogenases is highly specific for the presence, and correct positioning, of the nickel cation in the active site, which signifies completion of NiFe(CN)_2_CO cofactor insertion [55, 73–76]. Meanwhile, structures are available for several hydrogenase-specific endoproteases [55, 77–79] and all reveal a common αβα-fold structure, a similarity with peptidyl-tRNA hydrolases [79], but differences congruent with flexibility allowing the selectivity needed for the recognition of individual hydrogenase large subunits [20]. A common cleft within this family of protease harbours two conserved aspartate residues and a histidine residue that are proposed to coordinate the nickel ion. Notably, however, despite commonalities in residue conservation, these clefts show considerable variability in volume, which has been proposed to correlate with the respective residue number and size of the *C*-terminal peptide that needs to be cleaved [20].

Unexpectedly, it was the structure of the complex between the *T. kodakarensis* apo-hydrogenase large subunit HyhL and HypA [66] that provided the greatest insight into how both the *N*- and *C*-terminal peptides of the immature large subunit function to maintain the active site in an open conformation. The cleavage site on the *C*-terminal peptide of the hydrogenase large subunit is proposed to be initially shielded from the endoprotease by the formation of a new, transient interaction between the β11-strand with a β2-strand near the *N*-terminus. This frees up the *N*-terminal peptide of HyhL for interaction with HypA [66]. After nickel has been inserted, the β2 and β11 strands separate, allowing access of the protease (see [20]). Consequently, the *N*-terminus of the large subunit plays a significantly more important role in cleavage of the *C*-terminal peptide than had been previously considered. Nonetheless, the importance of the *N*-terminus of the large subunit was also predicted by *in silico* modelling and molecular dynamic simulations done with the *Rhizobium leguminosarum* HupL large subunit [80]. In their study, the authors predicted the highly flexible *C*-terminal peptide to be intrinsically disordered and thus incapable of acting as a binding platform for the conserved HypC or HypA proteins. This is also in agreement with the sequestered *C*-terminal peptide preventing the *C*-terminus-proximal active-site cysteine residue from only being able to close the bridge between the nickel and iron of the cofactor after *C*-terminal processing has taken place [45].

**Figure 3.**
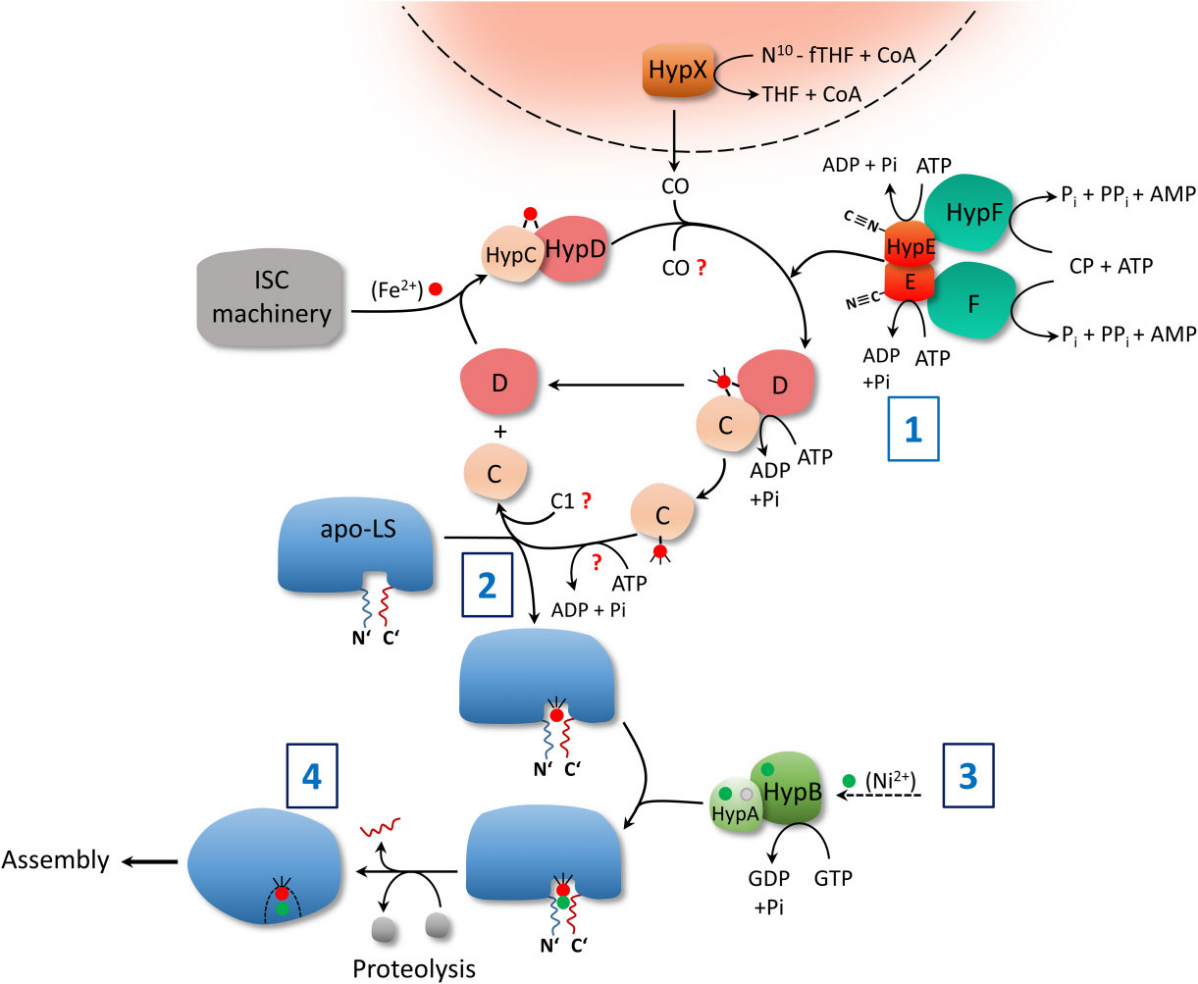
Schematic representation of the anaerobic biosynthesis and insertion of the NiFe(CN)_2_CO cofactor into the hydrogenase apo-large subunit. The involvement of HypX in generation of CO for carbonyl group attachment during hydrogenase maturation of O_2_-tolerant hydrogenases is depicted at the top of the figure with a dotted line and shading. Question marks signify unresolved biochemical processes. C1 indicates a carbon source originating from C1 metabolism. The boxed numbers correlate with the corresponding biochemical processes detailed in the main text.

Strict coordination of events in the insertion of the NiFe(CN)_2_CO cofactor is paramount for the majority of [NiFe]-hydrogenases (Fig. 3). This is particularly evident for membrane-associated, or periplasmically-localized enzymes, which are translocated across the membrane in their fully folded and cofactor-loaded states by the TAT (twin arginine translocation) system [81]. Premature association of the small subunit, which carries the *N*-terminally located TAT signal-sequence, with the immature large subunit must be prevented. This is probably achieved for the majority of hydrogenase systems by the presence of the *C*-terminal peptide, bound by further hydrogenase-specific accessory proteins [82]. This also explains why genetic removal of the *C*-terminal peptide allows TAT-competent translocation of [NiFe]-cofactor-free hydrogenase oligomers [83, 84], but not when cleavage of the *C*-terminal peptide is prevented by amino acid-exchange [64, 76, 85]. Notably, the experiments described by Massanz et al. [76] clearly demonstrated that the large subunit of the *R. eutropha* soluble hydrogenase with a mutated *C*-terminal peptide, retained the peptide and had nickel in the active site.

As is often the case in Biology, the ‘exception proves the rule’ and not all [NiFe]-hydrogenases have a *C*-terminal peptide that is cleaved to indicate completion of cofactor insertion, yet they nevertheless form active hydrogenase enzymes [84]. These include group 2b and 2d H_2_-sensing hydrogenases, group 4c hydrogenases and the group 4e Ech, energy-converting hydrogenases [2]. In their study, Hartmann et al. used genetic methods to remove the *C*-terminal peptide of the HoxG subunit of the O_2_-tolerant membrane-bound hydrogenase (MBH) from *R. eutropha* and were able to generate a catalytically active and membrane-associated enzyme, albeit with slightly lower overall enzyme levels [84]. Clearly, therefore there is variability in how essential the *C*-terminal peptide is in the maturation process. Importantly, however, that study nicely highlights the importance of the *N*-terminus of the hydrogenase large subunit in the maturation process.

## Ancillary proteins in the maturation of O_2_-tolerant hydrogenases

Apart from the HypA-F proteins, genes encoding ancillary proteins involved in [NiFe]-cofactor biogenesis have been identified in operons in, or near, those encoding hydrogenases in a number of different Pseudomonadota (synonymous with Proteobacteria). These ancillary proteins include HoxL, HoxV, HupF, HupK, and HypX [86–89]. HoxL and HupF are members of the HypC family, whereby paralogues of which are frequently, but not always [89], found within a single bacterium or archaeon synthesizing more than one hydrogenase [18–20]. HoxV (in *R. eutropha*) and HupK (in *Rh. leguminosarum*), on the other hand, are unusual in that they are proteins showing amino acid sequence similarity with [NiFe]-hydrogenase large subunits [88, 90], although HoxV has been shown to display no enzyme activity [91]. HoxV interacts specifically with apo-HoxG, the large subunit of MBH in *R. eutropha*, carries the Fe(CN)_2_CO moiety, but lacks nickel, leading to the suggestion that it functions as an intermediate scaffold, receiving the Fe(CN)_2_CO moiety from HypCD, before delivering it on via HoxL to apo-HoxG. HupK of *Rh. leguminosarum*, in contrast, interacts with HypC and with HupF (HypC homologue) [89, 92], whereby HupF also interacts with pre-HupL, the hydrogenase large subunit [89]. Notably, HupF has an extended *C*-terminus not found in other HypC proteins and it has been shown that this *C*-terminal extension is crucial for the synthesis of an active hydrogenase enzyme under conditions of high O_2_ tension [89]. Based on these findings, it has been proposed that HupF–HupK (HoxL–HoxV) function to provide protection against O_2_ during synthesis of the Fe(CN)_2_CO moiety [92]. As some bacteria that have a HoxV (HupK) protein synthesize hydrogenase only anaerobically [91], this suggests that some members of the HoxV–HupK family might provide a protective, or transient, storage location for the Fe(CN)_2_CO moiety during its synthesis. Apart from these features, however, little else is known about the precise role of these fascinating proteins in [NiFe]-cofactor maturation, clearly warranting further research.

HypX was initially partially characterized in *Rh. leguminosarum* and *R. eutropha* in the second half of the 1990s, where it was shown that it is involved in [NiFe]-cofactor biosynthesis [15, 93]. The protein has two domains, with the *N*-terminal one bearing a *N*^10^-formyl-tetrahydrofolate (*N*^10^-fTH) motif, while the *C*-terminal domain shows sequence similarity with enoyl-CoA hydratases/isomerases [94]. A series of elegant studies confirmed unequivocally that HypX generates the CO ligand from *N*^10^-fTH when the bacterium grows under comparatively high O_2_ partial pressures [95–97]. In the most recent of these excellent studies [97], the Lenz group showed that the *N*-terminal domain of HypX catalyses the transfer of the formyl group from *N*^10^-fTH to coenzyme A, generating formyl-CoA as an intermediate, but which remains associated with HypX. Subsequent decarbonylation of formyl-CoA by the *C*-terminal domain of HypX releases CO, which acts as a direct, gaseous source of the CO ligand [27, 28, 96, 97] (Fig. 3). Substrate-channelling within HypX and direct transfer of CO to the iron ion on the HypCD complex are presumably the final steps, implying that the generation of CO under anoxic growth conditions is the step that is sensitive towards O_2_ and must occur by a different mechanism, or originate from a different metabolic source.

## Proposals for the anoxic generation of the CO ligand

CO provided exogenously and under anoxic conditions can be quantitatively incorporated into the [NiFe]-cofactor resulting in enzymically active hydrogenases [27, 28]. The *in vitro* demonstration that HypX can generate CO strongly suggests that when *R. eutropha* grows in the presence of O_2_ and induces synthesis of hydrogenase, then HypX likely provides the CO ligand to the Fe associated with HypCD [96, 97]. This finding clearly indicates that there are at least two distinct pathways leading to generation of the CO ligand. Three key questions in [NiFe]-cofactor biosynthesis remain unanswered: (i) What is the metabolic source of the CO ligand during maturation of hydrogenase under anoxic conditions? (ii) Which enzyme, or enzymes, are involved in generating the CO ligand? (iii) Which is added to the iron ion first, the CO or CN-ligands?

A study performed several years ago identified a sub-population of anaerobically isolated HypCD complexes that had features of an O_2_-sensitive Fe–CO group, apparently lacking CN^−^ ligands [98], suggesting that either a step involving anaerobic CO generation, or the Fe(I)–CO moiety itself is sensitive to oxidation. HypX-dependent hydrogenase maturation under oxic conditions [96] would be commensurate with an early step in maturation being O_2_-sensitive, particularly as HypCD carrying the synthesized Fe(CN)_2_CO moiety is insensitive to O_2_ [98]. If correct, this also supports the hypothesis that CO might be added to the iron before the two CN^−^ ligands. Chemically, however, either CO or CN^−^ could be added first (C. Pickett, personal communication).

The HypX study by Schulz et al. is also significant from the point of view of the enzyme’s substrate, *N*^10^-fTHF [97]. A formyl group can be readily reduced to CO by a suitable reductase activity. Notably, HypD also has an enoyl-CoA hydratase motif [52], which might suggest HypD could perform a similar decarbonylase reaction to that catalysed by HypX. The question then would be where the formyl-CoA substrate originates. Formyl-CoA can be generated from acetyl phosphate by a CoA transferase, or from formyl-phosphate by phosphotransacetylase [99, 100]. Like *Oxalobacter formigenes* [101], *E. coli* also has both an oxalyl-CoA decarboxylase (YfdU) and a formyl-CoA transferase (YfdW) [102], which could act as a source of formyl-CoA. Interestingly, formyl-phosphate can also be generated in an ATP-dependent side reaction of CP synthetase [103], providing a potential alternative route to formyl-CoA.

Apart from a formyl intermediate, the other most likely candidates as a source of CO are either formate or CO_2_. Labelling studies have ruled out exogenously added CO_2_ functioning as a direct source of the CO ligand in *R. eutropha* (*C. necator*) [28]. Nevertheless, as a mutant specifically defective in CO-ligand synthesis has yet to be described, it is conceivable that more than one metabolic source for the generation of the carbonyl ligand exists in many microorganisms. Titration experiments performed by Bürstel et al. [95] demonstrated for *R. eutropha* regulatory hydrogenase that an exogenous carbon monoxide concentration of around 10 nM was sufficient to outcompete the endogenous CO precursor. Considering that formyl groups, intracellular CO_2_ generation by decarboxylation reactions, or formate abound in microorganisms, limited amounts of one of these substrates will always be available.

**Figure 4.**
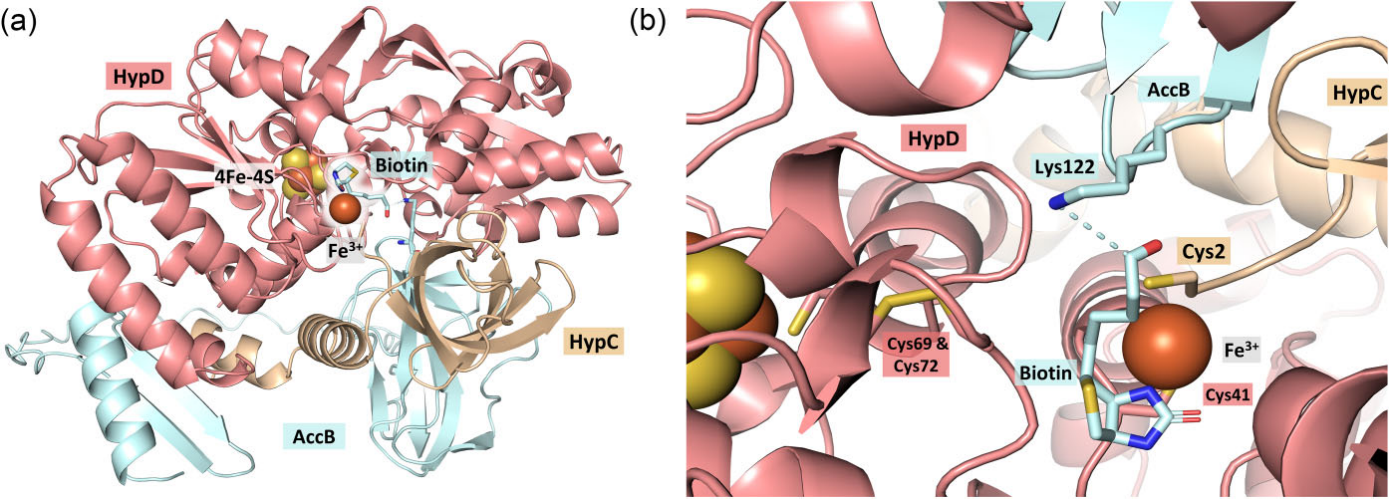
Shown is an AlphaFold3 simulation of *E. coli* HypCD with AccB (a). The ipTM and pTM scores of the structure are 0.71 and 0.76, respectively. When the first 80 *N*-terminal amino acids of AccB are omitted, the ipTM and pTM scores improve to 0.85 and 0.88, respectively. A Fe^3+^ ion was simulated together with the proteins. AlphaFill was used to add automatically the [4Fe–4S]-cluster and the biotin aldehyde into the structure. (b) Depiction of the region around the predicted binding site of the Fe^3+^ ion is shown. The locations of the key cysteine residues of HypC and HypD are shown and the dashed line shows the expected *in vivo* chemical bond of the biotin to the lysine 122 of AccB.

Finally, during the purification of the HypCD complex from *E. coli* extracts, we have consistently identified the biotin-carboxyl carrier protein (AccB) component of acetyl-CoA carboxylase [104] as a ‘contaminant’, despite stringent controls ruling out AccB binding non-specifically to the chromatography columns used (S. Müller, D. Lubek, G. Sawers unpublished observations). This observation was ignored for many years until the development of the AlphaFold3 structural simulation algorithm [105], revealed a surprisingly good interaction of AccB with the HypCD complex (Fig. 4a). Indeed, upon closer inspection of the structure, the predicted site of CO_2_-binding to the biotin is positioned directly within hydrogen-bonding distance to the Fe ion predicted to be coordinated by C2 of HypC and C41 of HypD (Fig. 4b). It has been previously suggested that the other possible function of the ATPase activity of HypCD is to provide the thermodynamic driving force to dehydrate CO_2_ to CO [52].

## Unresolved aspects of [NiFe]-hydrogenase maturation

The metabolic origin of the anaerobically generated carbonyl ligand of the [NiFe]-cofactor needs to be determined. Based on the studies carried out to date, it appears clear that it probably originates from C1 metabolism, in analogy to what has been clearly demonstrated for CO synthesis in aerobes [97]. Nevertheless, it is also likely that more than one metabolic intermediate can act as a precursor and this might also differ between microorganisms that synthesize [NiFe]-hydrogenases. The key issue to be resolved is whether, as proposed for HypX, a decarbonylase-type reaction releases CO locally for attachment to the iron ion in HypCD, or whether HypCD itself catalyses conversion of a precursor metabolite that is already liganded with the iron ion, thus obviating CO release. This question is also very relevant to address with respect to defining any additional role ATP might have, and with respect to the order-of-addition of the diatomic ligands.

The resolution of the structure of HypCD with an attached Fe(CN)_2_CO moiety would be a major coup and attempts to obtain structural information on such a complex in the presence of ATP might prove fruitful. Furthermore, despite the demonstration that HypCD isolated from an *E. coli carAB* mutant lacks any indication of CO or CN signals in infrared spectroscopy [35], a further analysis of HypCDE complexes might prove to be more revealing, especially as the proposed intermediate with only CO attached to an Fe (I) species is highly labile [98]. Structural analyses analogous to those that defined the HypA–HyhL [66] structure will be very informative regarding how the transfer of the Fe(CN)_2_CO moiety from HypC into the apo-large subunit occurs.

Regardless of whether HypCD is involved in direct or indirect carbonyl-ligand generation, the source of the electrons for the reduction of the [4Fe–4S] cluster in HypD is still unknown. Ferredoxin can be ruled out as electron donor to HypD of *E. coli* (C. Kulka, A. Haase, G. Sawers and I. Zebger unpublished data) and an alternative electron donor could be one of the two flavodoxins in the bacterium. Finally, defining the role of the HoxV ancillary proteins that are found in many bacterial species is important, in particular regarding what evolutionary advantage this class of ‘Fe(CN)_2_CO-storage’ proteins might provide in the cofactor maturation process.

## Data Availability

The data underlying this article are either available in the article or will be shared on reasonable request to the corresponding author.
